# Quantifying cell death induced by doxorubicin, hyperthermia or HIFU ablation with flow cytometry

**DOI:** 10.1038/s41598-021-83845-2

**Published:** 2021-02-23

**Authors:** Paul Christopher Lyon, Visa Suomi, Philip Jakeman, Leticia Campo, Constantin Coussios, Robert Carlisle

**Affiliations:** 1 Nuffield Department of Surgical Sciences, John Radcliffe Hospital, University of Oxford, Oxford, OX3 9DU UK; 2 Institute of Biomedical Engineering, University of Oxford, Old Road Campus Research Building, Oxford, OX3 7DQ UK; 3 Department of Oncology, University of Oxford, Old Road Campus Research Building, Oxford, OX3 7DQ UK

**Keywords:** Biomedical engineering, Apoptosis, Translational research, Computational models, Flow cytometry, Drug delivery, Cellular microbiology

## Abstract

Triggered release and targeted drug delivery of potent anti-cancer agents using hyperthermia-mediated focused-ultrasound (FUS) is gaining momentum in the clinical setting. In early phase studies, tissue biopsy samples may be harvested to assess drug delivery efficacy and demonstrate lack of instantaneous cell death due to FUS exposure. We present an optimised tissue cell recovery method and a cell viability assay, compatible with intra-cellular doxorubicin. Flow cytometry was used to determine levels of cell death with suspensions comprised of: (i) HT29 cell line exposed to hyperthermia (30 min at 47 °C) and/or doxorubicin, or ex-vivo bovine liver tissue exposed to (ii) hyperthermia (up to 2 h at 45 °C), or (iii) ablative high intensity FUS (HIFU). Flow cytometric analysis revealed maximal cell death in HT29 receiving both heat and doxorubicin insults and increases in both cell granularity (p < 0.01) and cell death (p < 0.01) in cells recovered from ex-vivo liver tissue exposed to hyperthermia and high pressures of HIFU (8.2 MPa peak-to-peak free-field at 1 MHz) relative to controls. Ex-vivo results were validated with microscopy using pan-cytokeratin stain. This rapid, sensitive and highly quantitative cell-viability method is applicable to the small masses of liver tissue typically recovered from a standard core biopsy (5–20 mg) and may be applied to tissues of other histological origins including immunostaining.

## Introduction

In the clinical use of focused ultrasound (FUS) or high intensity FUS (HIFU) devices for conventional cancer ablation in solid organs, localised coagulative necrosis is the desired end result. As with any ablative modality, for the best prognostic outcomes it is crucial that near-complete cell death is achieved to adequate margins to avoid local tumour progression^1^.

In addition to traditional ablative regimes, HIFU devices may also be used at lower pressures to induce localized hyperthermia, for example to deliver and/or enhance the effect of chemotherapeutics. It has been understood for some decades that, when combined with specific chemotherapy agents, regional mild hyperthermia of 40–43 °C has a tumour-selective synergic cytotoxic effect without affecting systemic toxicity in vivo^2,3^. Such combination therapy has demonstrated enhanced cytotoxic effect clinically with a range of chemotherapeutics and may be supra-additive, often with greatest effect when the two treatments are applied simultaneously. Suggested mechanisms include increased drug uptake, increased alkylation and inhibition of DNA repair^4^.

Recently the lyso-thermosensitive liposomal doxorubicin (LTLD) formulation (ThermoDox), a thermally activated liposomal drug delivery system developed under Celsion’s investigational drug program, has demonstrated relative stability at body temperature (37 °C) allied to the capacity to release free drug at temperatures greater than 39.5 °C^5,6^. Such formulations may be ideal oncological drug-delivery systems when used in combination with FUS-mediated mild tumour hyperthermia (increases of around 3–5 °C), thereby achieving targeted intratumoral drug release. This approach relies on sub-ablative levels of hyperthermia and methods of validating the mechanism of subsequent cell death are great importance in early studies; significant over-heating will cause direct tissue ablation, as with conventional HIFU, rather than desired chemo-ablation due to localized release from LTLD. Indeed, early ablation may even damage tumour vasculature to the extent that it can no longer support supply therapeutic levels of LTLD to the tumour.

**Figure 1:**
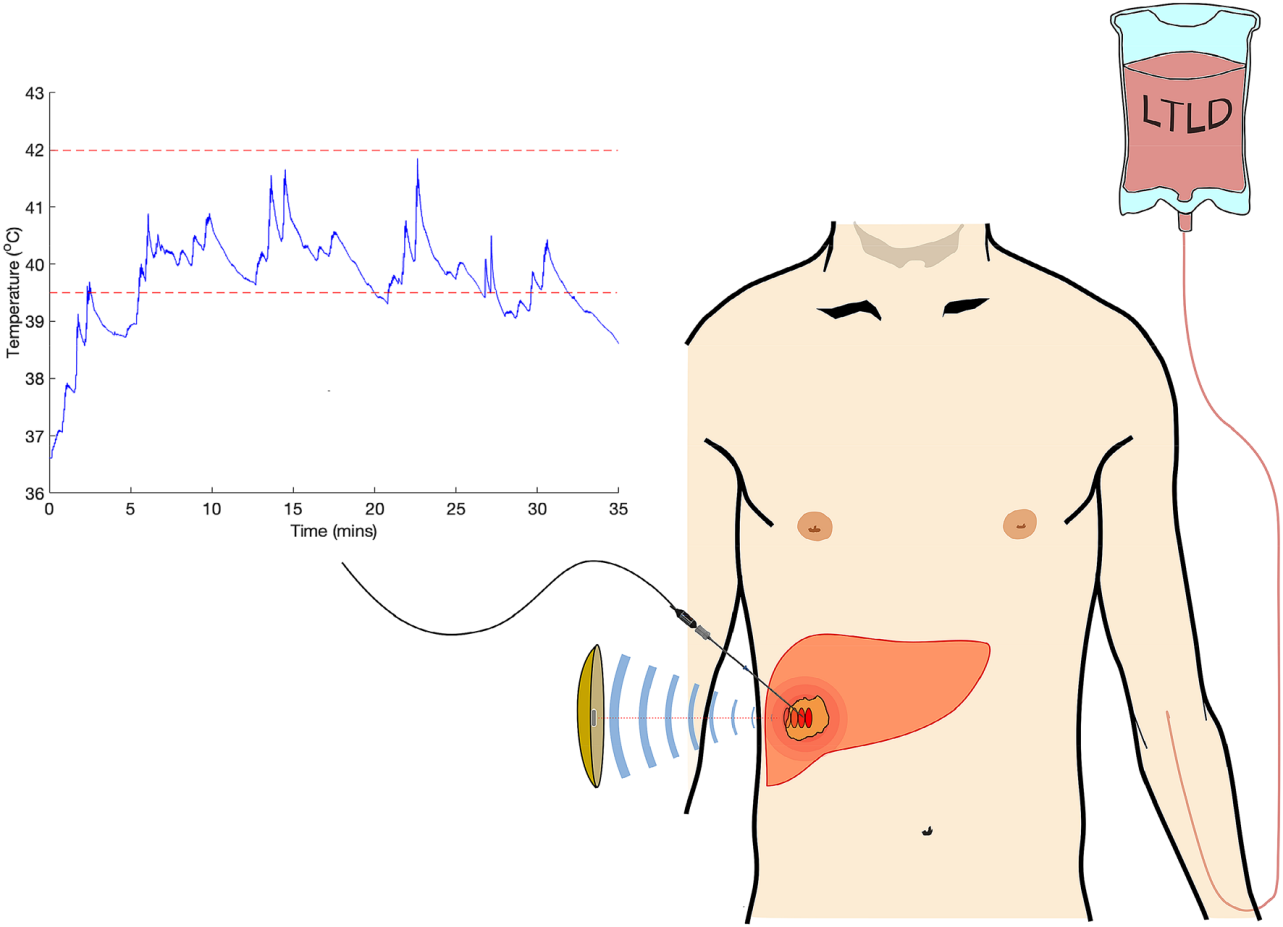
Schematic depicting the use of an extracorporeal USgFUS device (JC-200, Chongqing Haifu Medical Technology Co., Ltd.) and implanted thermistor for targeted LTLD delivery to liver tumours in the TARDOX study, Oxford, UK. RIGHT: LTLD was infused intravenously prior to FUS and biopsies of the target tumour were taken before and after infusion and finally after FUS exposure. LEFT: illustrative thermistor trace obtained for the first patient treated, demonstrating the approximate range of sub-ablative levels of hyperthermia sought (39.5–42 °C) centrally within the target tumour.

Real-time monitoring of in vivo temperature changes using clinical MR- (MRgFUS) or ultrasound-guided (USgFUS) FUS devices is a non-trivial problem and there are many active areas of theoretical and pre-clinical research in this regard^7^. With advances in MRI-thermometry, non-invasive targeted drug delivery using FUS for hyperthermia has gained renewed interest and is under investigation both pre-clinically and clinically. Many pre-clinical studies have demonstrated feasibility, safety and efficacy of this delivery mechanism, including studies by Staurch et al*.* in a rabbit tumour model using an MRgFUS device^8–10^. Other clinical studies, namely the HEAT and OPTIMA studies, have focused on use of LTLD in combination with radiofrequency ablation (RFA) in order to achieve enhanced cytotoxic effect at the hyperthermic ablation margin^11^. More recently the TARDOX study, a first-in-man Phase I clinical study at Oxford, UK, has demonstrated safety, feasibility of efficacy of LTLD delivery to liver tumours using USgFUS. In the first phase of the study, an implanted thermistor was used for real-time thermometry (Fig. 1) and subsequently predictive computational models were used to determine power settings for hyperthermia consequent with non-invasive drug delivery^12–14^. In use of FUS devices for targeted drug delivery by hyperthermic rather than ablative regimes, lower pressures may be employed naturally affording improved safety profiles and minimizing off-target effects. Furthermore, there is greater potential for eventual deployment of smaller portable and possibly hand-held FUS heating devices.

The aim is targeted mild hyperthermia for localised delivery of a high concentration of cytotoxic drug at powers below the threshold for instantaneous ablative cell death. Based on a modified Arrhenius-based system, early work by Sapareto and Dewey^15^ lead to introduction of the concept of a thermal isoeffect dose (TID) model defined by Cumulative Equivalent Minutes at a reference temperature of 43 °C (m)^16–18^. By integrating a thermal dose profile over time and normalizing to an equivalence dose at the reference temperature, the model has been used to predict cell death. The CEM_43_ model was first applied to HIFU ablations in vivo by Damianou et al.^19^ and was later used clinically to quantify hyperthermia-enhanced radiation response in superficial tumours^20^. The model has become the industry standard for ablative systems^21^ and most recently for MRgFUS ablation of fibroids with accurate histopathological correlation^22^. However, the model was primarily developed for hyperthermia applications and validated in a subset of human in vitro cell lines up to 50 °C and for gradual temperature rises only. However the thermal dose required to cause cell death can vary considerably across different cell lines^23^. Furthermore, the model does not include the effect of increased perfusion at low thermal dose and stasis at high thermal doses^24^. It has not been validated in vivo for tissues exposed to instantaneous temperature rises above 50 °C, i.e. those which occur during ablative HIFU. Dewhirst et al. identified the lack of application of the model to human tissues^25^. Further complexity is introduced clinically by the range of histological tumour subtypes, varying microregional degrees of perfusion and anatomical differences, for example the presence of overlying acoustically blocking ribs. Whilst the CEM_43_ model can provide a simplified overall thermal dose metric, it should not be relied upon to predict presence or absence of coagulative necrosis by FUS, which is a distinct concept. FUS-induced tissue damage may be induced by a combination of mechanisms including mechanical damage achieved through cavitation, direct thermal ablation or cumulative thermal dose^26^. Thus, coagulative necrosis may be achieved without hitting the CEM_43_ threshold^23^, and conceivably the converse may also be true. Hence, robust methods for quantifying cell viability of tissues, such as liver tumours and healthy liver hepatocytes, following targeted hyperthermia (or ablation) would be advantageous to further validate predictions from the CEM_43_ model and other thermal models.

During early-phase clinical studies investigating targeted drug-delivery, a biopsy or tumour sample obtained following targeted hyperthermia-mediated drug delivery may be utilised for purposes of drug quantification^14^. Dependent on tissue availability, there may be utility for a robust quantitative method for assessing cell viability in samples obtained following FUS exposure. Using samples obtained shortly following FUS exposure, such a method could be used to validate that subsequent radiological response is due to chemo-ablation rather than instantaneous thermal ablation^12^.

Immunohistochemical staining for microscopy has typically been used to assess clinical samples for cell necrosis following targeted ablation of tumours^27–29^. In the TARDOX study, ultimately microscopy with immunohistochemistry was used to analyze tumour biopsy samples and the presence of CK-8 staining was used to demonstrate that tumour cells exposed to FUS remained viable shortly following exposure, as assessed by a histopathologist^12,30^. Microscopic analysis was chosen as it has the advantage of providing information about the tumour morphology, vascular supply and doxorubicin distribution and could be performed on the very limited tissue masses remaining (around 2 mg) after drug quantification studies using high performance liquid chromatography (HPLC). However, microscopic examination of tissues for cell viability is essentially a qualitative technique and may suffer from assessor subjectivity. Furthermore, high auto-fluorescence of background liver tissue and the fluorescence of study drugs such as doxorubicin make cell viability studies using fluorescent probes more challenging.

Flow cytometry is a well-established technique that has been used to quantify cell death, apoptosis and other characteristic effects in various cell lines exposed to different hyperthermia regimens in several in vitro studies^31–46^. One in vivo study has utilized flow cytometry to determine DNA content of spermatocytes recovered from the testes of mice following hyperthermia using an alternative cell recovery technique^47^. Another study has looked at response in leukocytes recovered from mice undergoing whole-body hyperthermia^48^, subsequently studied clinically^49^. The versatility of flow cytometry is demonstrated by many other studies, including two which have used it to quantify intracellular^50^ and nuclear accumulation of doxorubicin^51^ in different cell lines.

In light of emerging early phase clinical studies employing hyperthermia mediated FUS for targeted drug delivery, we have explored flow cytometry as an alternative technique to quantify cell death in tissues exposed to FUS. We present a tissue cell-recovery method and flow cytometry viability assay, developed with the aim of distinguishing ablative versus sub-ablative levels of tissue damage in biopsy-sized tissue masses. The method has been validated by in vitro HT29 cell cultures exposed to hyperthermia and/or doxorubicin and freshly harvested ex-vivo bovine liver exposed to sub-ablative or ablative levels of focused ultrasound. It is likely that the method presented is applicable to pre-clinical and clinical samples including solid tumours exposed to hyperthermic and ablative modalities. In particular the assay was designed to be practical for tissue masses of as little as 5 mg, (i.e. as obtained with an 18G core biopsy device^52^), and to be compatible with the presence of a study drug, doxorubicin. Doxorubicin is one of the most commonly used chemotherapeutic agents in pre-clinical clinical studies, due to the inherent fluorescence of the molecule making it an excellent theranostic agent. However, it has a broad emission spectrum^53^, which is known to interfere with a number of commonly used fluorophores used for the assessment of cell viability by flow cytometry, including propridium (PI) iodide^54^. In particular, doxorubicin typically has an excitation wavelength of ~ 480 nm with a peak emission at ~ 590 nm, overlapping the excitation (400–600 nm) and emission spectra for PI (600–700 nm), although exact values are somewhat solvent-dependent^53,55^. For PI an up to 30-fold dynamic range of weak staining of viable cells and strong staining of non-viable cells can be expected^56,57^.

At its most fundamental level, flow cytometry utilises the light scatter from the intersection of the laser beam light to characterise both cell size and cell structure. Forward scatter (FSC) correlates to light scattered in the forward direction, being directly related to the cell size. Side scatter correlates to light scattered in the perpendicular plane and is affected by the cell’s refractive and reflective indices. Increase in side scatter (SSC) may be due to inhomogeneity within the cell morphology, for example condensation of the cytoplasm or nucleus, or increased cell granularity^58^. In early apoptosis cells demonstrate transient increase in SSC (inhomogeneity). SSC markedly decreases in late apoptosis when the cell also condenses, with and associated marked decrease FSC (cell size)^58,59^. SSC, when used in combination with FSC, can also be used to distinguish cell type, for example granulocytes have a high SSC relative to lymphocytes.

## Materials and methods

### Cell viability stains for flow cytometry

Flow cytometric analysis was performed using a BD FACSCalibur flow cytometer. Cells were either stained with propidium iodide (PI) using published methods^60^, or a commercial far-red fluorescence cell viability kit (LifeSciences Far Red LIVE/DEAD Fixable Far Red Dead Cell Stain Kit for 633 or 635 nm excitation, Ref: L10120 (L/D stain)). The L/D stain was selected due to its far-red fluorescence emission spectra being distinct from that of doxorubicin (excitation maximum of 633 nm and emission maximum of 655 nm).

Cells stained with PI were excited using the blue (argon) laser at 488 nm and its broad-emission spectra was detected using the orange detector (585 ± 42 nm) on the FL2 channel. Cells stained with the L/D stain were excited using the far-red laser (633 nm) and its narrow emission spectra was detected using the far-red detector (660 ± 16 nm) on the FL-4 channel, in logarithmic 4-colour mode. Up to the first 10,000 events were used for analysis, discarding any events with forward scatter (FSC) less than 100 (presumed to represent cell debris and/or impurities).

The PI stain is a cell-impermeant dye that is excluded from viable cells with excitation and emission maxima of 535 and 617 nm with weak fluorescence on the surface of cells. Once a cell membrane has been compromised and PI can enter the cell, it intercalates with DNA, enhancing its florescence up to 30-fold^56,57,60^.

Increased levels of L/D staining are indicative that the fluorescent reactive dye has permeated damaged cell membranes to react with intra-cellular amine groups and therefore that cell viability has been compromised. This is in contrast to weak levels of L/D staining which occurs in viable cells for which only cell surface amine groups are available for reaction due to membrane impermeability. Viability staining was performed as described and samples were either assayed or fixed with within 60 min of staining.

For each stained sample a matched unstained control of equivalent volume and cell concentration was used. 1 μL of defrosted L/D stain (previously reconstituted in dimethyl sulfoxide (DMSO) and stored in aliquots of 5 μL at 80 °C) was added to samples for staining only and gently vortexed. Unstained and stained samples were incubated for 15 min at room temperature and protected from light. Centrifugation was repeated and the pellets re-suspended in 1 mL of 4% NBF diluted in DPBS (Scigen 10% Buffered Neutral Formalin, Ref: FX2040) in an incubator for 2–4 h at room temperature, protected from light to fix the stain. Further centrifugation was performed with resuspension in 650 μL of DPBS, then aliquot into two 300 μL sterile lidded FACS tubes (one spare) and refrigerated covered in foil for subsequent analysis within a few days.

### Flow cytometric assessment of cell viability using HT29 cell suspension exposed to doxorubicin and/or hyperthermia

Cell suspensions of the HT29 colorectal cancer cell line (American Type Culture Collection, ATCC HTB-38) were grown in DMEM (High glucose Dulbecco’s Modified Eagle Medium, LifeTechnologies Ref: 11965-092) with 10% FCS (HII Foetal Calf Serum, LifeTechologies, Ref: 10500-064) and 1% Penicillin–Streptomycin (LifeTechnologies, Ref: 15140-122) in 250 cm^2^ culture flasks. Cells were trypsinised into a suspension, centrifuged, trypsin was removed, and cells were re-suspended in 10 mL of the same cell media. Following a gentle vortex, the cell suspension was split four ways into Falcon tubes, two receiving 1 mg/mL doxorubicin solution (Doxorubicin, 100 mg, Apollo Scientific, Ref: BID0120, Batch No: AS444828), made up in Dulbecco’s phosphate buffered saline (DPBS, no calcium, no magnesium, LifeTechnologies, Ref: 14190-250) up to a final concentration of 100 μg/mL, and two DPBS alone. One doxorubicin-spiked tube and one DPBS sample were then heated to 47 °C in a water bath for 30 min (confirmed by reference to temperature-measured control tubes), and the remaining two samples placed in a CO_2_ incubator at 37 °C. Shortly following hyperthermia or normothermia, the contents of each of the four Falcon tubes were divided between four different 5 mL sterile fluorescence-activated cell sorting (FACS) tubes (BD Falcon round-bottom polystyrene tubes, Catalog No. 352058). The 16 FACS tubes were protected from light and kept in a CO_2_ incubator at 37 °C when not being assayed.

Cell viability was assessed using flow cytometric analysis, as detailed below, at the 2, 6, 24, 48 and 72-h following initial doxorubicin exposure (or not). At each time point, analysis of the four corresponding samples (DPBS/37, DPBS/47, Dox/37, Dox/47 °C) was completed within 90 min of being taken out of the incubator without a fixation step. Contents of the FACS tubes were rescued by trypsin treatment prior to assay to prevent adherence of cells to the FACS tubes. First, existing media was pipetted into a fresh tube. 500 μL of trypsin (1:10 Trypsin (10X) LifeTechnologies, Ref: 15090-046) diluted in serum-free DMEM was then added to the remaining cells and tubes were incubated at 37 °C for 15 min, and, after mixing with a pipetting action, remaining content was transferred to the corresponding receiving tube used for immediate viability staining and flow cytometric analysis (see below).

### Method of liver tissue processing to produce cell suspension

Ex vivo mouse liver was obtained from recently culled control mice from an unrelated study performed in accordance with relevant animal licensing, guidelines and regulations. No live mice were used in the generation of data for this manuscript. The cell recovery protocol involving tissue disaggregation and enzymatic digestion was first optimised using small (from 4 to 100 mg) samples of ex-vivo fresh mouse liver. Post-treatment tissue samples were placed in individual petri dishes, covered by a few drops of sterile DMEM and then kept on ice. Tissue was macerated between two crossed scalpels into a pulp, avoiding crushing and scraping. Samples were washed in up to 10 mL of DMEM media and transferred to 15 mL centrifuge tubes. 200 μL of 1 mg/mL Collagenase/Dyspase diluted in DPBS (100 mL, Roche, Ref: 10269638001) was added for digestion of the connective tissues, vortexed and incubated in a water bath at 37 °C for 30 min. Samples were filtered through cell strainer (BD Falcon Cell Strainer 70 μm nylon mesh, Ref: 352350) into a fresh 50 mL centrifuge tube. 2 mL of RBC lysis buffer diluted in deionized water (Red Blood Cell Lysis Buffer, 100 mL, Sigma, Ref: R5575) was added and each sample kept on ice for 5 min. Samples were centrifuged at 400×*g* (RCF) at 10 °C for 5 min and the supernatant was discarded to leave pellet (not visible by eye for smaller mass samples). Cells were re-suspended in 5 mL FACS buffer (2% foetal calf serum in DPBS), re-filtered through a fresh cell strainer into a centrifuge tube and topped up to 15 mL with FACS buffer. Centrifugation was repeated and cells were re-suspended in 2.1 mL DPBS and vortexed. 1 mL aliquots were transferred into two 15 mL centrifuge tubes (one for staining, one unstained control). For each sample, cell counts were performed using haemocytometer and, in the case of larger tissue masses, diluted in DPBS to approximately 1 million cells/mL if exceeding this concentration.

### Flow cytometric assessment of cell viability method using ex-vivo liver tissue exposed to hyperthermia

The liver of a freshly slaughtered ox was obtained and a small peripheral tissue block of 5 × 5x10 cm was excised. From this section 18 smaller single-piece sections were cut into similar sizes with masses ranging from 71 to 130 mg. Each section was placed in a ZipLock polyethylene bag and immersed in 4 mL of Roswell Park Memorial Institute (RMPI) medium. Air was expelled before sealing and applying custom-made Delrin clamps to immerse the bags over a water bath. Thermocouples (Type T Thermocouple, Omega, Ref: HYP-0-33-1-T-G-60-SMPW-M) were placed through modified clamps of the longest duration control and hyperthermia samples. The thermocouples were inside the bags, passing through the media with tips just within the liver tissue. All bags were watertight and all but three were loaded onto a custom-made holder, which was immersed in a 45 °C water bath. The remaining three bags were placed into a 37 °C water bath. Bags were removed from the hyperthermic water bath in triplicates at 15, 30, 60, 120 and 240 min, and placed into the 37 °C bath along with the controls. Samples were recovered as per the cell recovery protocol, with the exception of incubating the resulting suspensions overnight in a CO_2_ incubator without fixation prior to analysis, to allow time for the effects of heat insult to manifest. The samples were removed from the incubator 16-h post-recovery, stained with L/D stain and formalin-fixed for same-day flow cytometric analysis.

### Flow cytometric assessment of cell viability using ex-vivo liver tissue exposed to HIFU ablation

**Figure 2:**
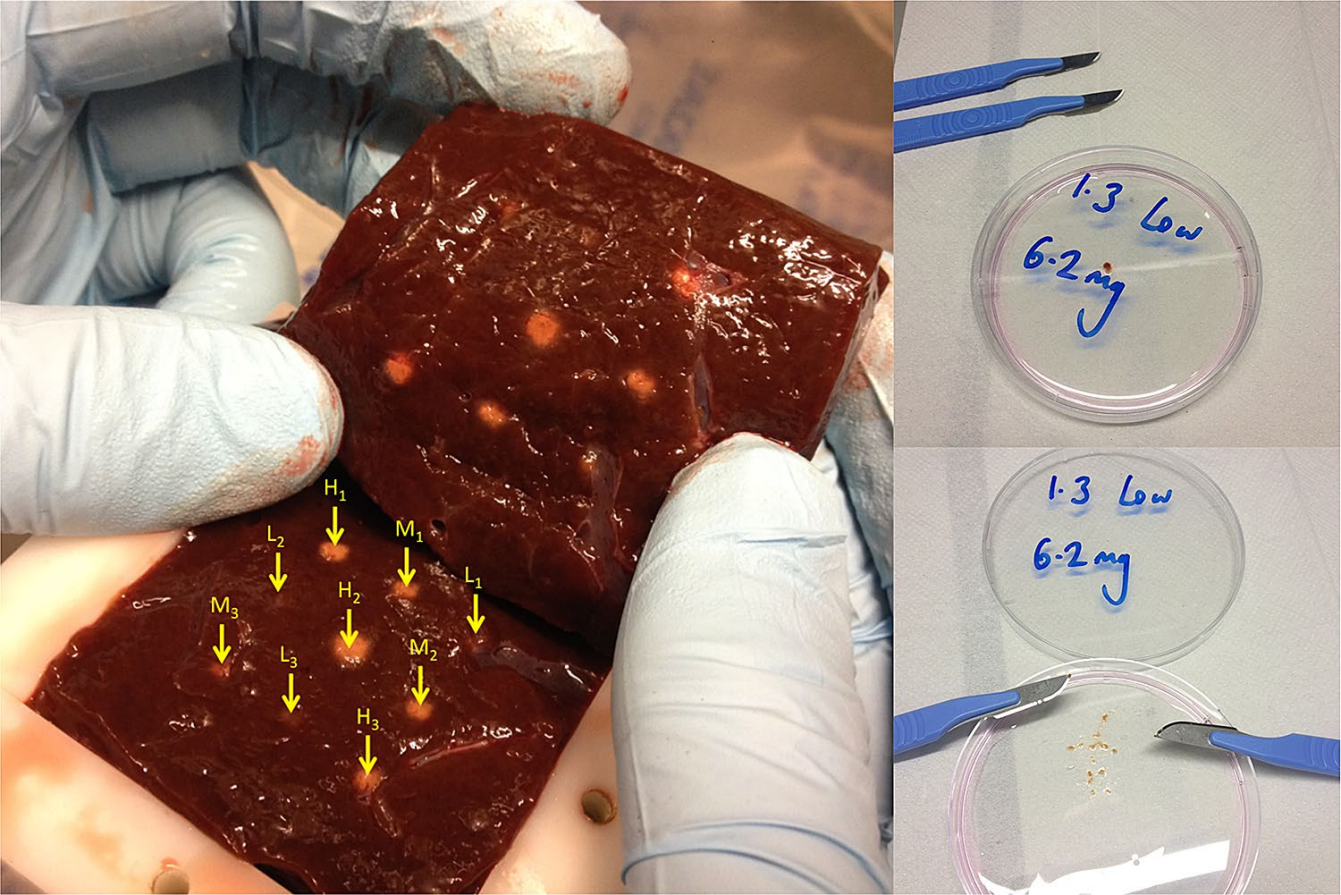
Experimental images of ablated *ex-vivo* bovine liver. LEFT: N = 3 HIFU lesions for each of H = High = 8.2 MPa (peak-to-peak), M = Medium = 7.3 MPa and L = Low = 6.3 MPa acoustic pressures obtained in freshly harvested degassed ex vivo bovine liver. Liver tissue remains in watertight tissue holder with the acoustic window removed and has been sliced transversely at the axial focus (2 cm depth). Lesions for each pressure regime are staggered across rows to help locate the (more subtle) low power lesions relative to the other lesions. RIGHT: A biopsy sample of 6.2 mg liver mass exposed to low HIFU pressures obtained from the L1 lesion before (TOP) and after (BOTTOM) physical disaggregation using crossed scalpels in petri dish, prior to enzymatic digestion.

Using a custom watertight tissue holder with internal dimensions 5 × 5 × 10 cm, liver obtained from a freshly slaughtered ox was cut to size to fill the holder and degassed in DPBS. The ex vivo liver was exposed to HIFU through a 5 × 5 cm acoustic window (Mylar sheet) at an axial tissue depth of 2 cm in a degassed water tank at 22 °C. Using a 1.067 MHz transducer at the fundamental and a custom 3-axis positioning system, three lesions were created for each of three selected pressures (Low = 6.34 MPa, Medium = 7.27 MPa and High = 8.19 MPa peak-to-peak acoustic pressure, free-field). To increase the cross-sectional and thus sampling area for each lesion, rather than increasing duration of sonication, each of the individual nine lesions were formed using 10 s continuous wave exposures arranged in a 2 × 2 grid with 1 mm separation in both X- and Y-axes. The nine (3 × 3) resultant lesions (each consisting of 4 sub-lesions) had a 1 cm separation in the X- and Y-axes (Fig. 2).

The core of each lesion was biopsied individually such that the analysis could be performed in triplicate, in addition to three controls taken from non-ultrasound exposed regions. The cell recovery protocol was performed with fixation within 4 h of HIFU exposure for all lesions. Fixation was performed within this short post-recovery period to avoid ensuing cell death due to removal of the liver tissue from its blood supply. The following day, resulting cell suspensions were assessed for yield and viability using the flow cytometric protocol.

### Immunohistochemical methods using ex-vivo liver tissue exposed to HIFU ablation

Using the methodology described above further *ex-vivo* liver tissue was exposed to HIFU ablation at high pressure (8.2 MPa peak-to-peak), over the same 2 × 2 ablation grid but with longer 40 s HIFU exposures. Within one hour of HIFU exposure, 2-3 mm cross-sections of the ablated and control zones, were then resected from the liver sample, and formalin-fixed and paraffin embedded for histopathological correlation, using standard laboratory protocols. Samples were sectioned and stained for light microscopy using both the wide-spectrum pan-cytokeratin (pan-CK, Anti-wide spectrum Cytokeratin antibody ab9377, Abcam), an immunohistochemical stain which stains the vital parenchymal and bile duct cells of the liver cytoskeleton^61^, but whose stain is lost in non-viable cells, for example post-ablation^30^, and haematoxylin and eosin (H&E) using standard laboratory protocols.

### Method for simulation of HIFU exposure

Previous pre-clinical studies have shown that simulations combining Pennes’ bioheat transfer (BHT) equation^62^ with mathematical focused ultrasound models, can be used to closely predict temperature rises and presence of ablative lesions in vitro using HIFU exposure parameters and depth of tissue penetration. One study used ex vivo bovine liver to demonstrate close correlation between such a mathematical model using a very similar clinical HIFU device by the same manufacturer (Model-JC, Chongqing Haifu Medical Technology Co., Ltd)^63^. A more recent study has used an non-linear model to predict HIFU-induced temperature rises in ex vivo porcine muscle experimentally using MRI thermometry, with excellent correlation for peak temperatures below 85–90 °C in three dimensions^64^.

In the present study, the simulations of the pressure fields and the temperature evolution during the sonications in ex vivo and non-perfused liver were conducted in Matlab R2013b (MathWorks Inc, Natick, MA, US) using a non-linear HIFU simulator^65^. The simulation model used in has been experimentally validated having the variation up to 10% in acoustic pressure and up to 20% in temperature^66^. This variability is largely accounted for the uncertainties in measuring the acoustic and thermal properties in tissue, and thus, based on published literature, a range of liver tissue attenuation properties were included in the analysis when conducting the simulations. Therefore, the simulation results in this study can be seen as useful references for the temperature ranges which can be expected in real liver tissue when estimating sonication parameters in similar studies.

First the ultrasound pressure fields were computed as spatial pressure distribution of a spherical transducer by solving the axisymmetric Khokhlov–Zabolotskaya–Kuznetsov (KZK) equation^67,68^ in the frequency domain. The solution takes into account beam diffraction, non-linear effects^69^, wave reflection at interfaces, phase speed dispersion in the medium and the frequency dependence of absorption. The non-linearity of the pressure fields and liver were taken into account using 128 harmonic frequency components and typical non-linearity parameters for both liver and water^70^. The computed free-field peak-to-peak pressure values were in close agreement with the corresponding values of 6.34, 7.27 and 8.19 MPa measured experimentally using a hydrophone. Following derating for liver attenuation, the pressures were 6.47, 6.10 and 5.68 MPa peak-to-peak at low pressure, 7.54, 7.10 and 6.60 MPa at medium pressure and 8.38, 7.89 and 7.33 MPa at high pressure for low, medium and high liver attenuations respectively. Note that the peak-to-peak pressure values appear higher than in water in the case of low attenuation liver, which is due to non-linearity and refraction effects^69^.

After solving the pressure fields, the temperature evolution during a continuous 10 s sonication and the 2-D spatial distribution in the end of the sonication were computed by solving the Pennes’ bioheat transfer (BHT) equation^62^. Because the liver was used ex vivo in the experiments, the perfusion term of the BHT equation was set to zero. The attenuation of the liver was varied between low (0.14 dB/cm/MHz^0.84^), average (0.39 dB/cm/MHz^1.11^) and high (0.67 dB/cm/MHz^1.49^) values found in the literature^71,72^ to illustrate the effect of varied attenuation on temperature conversion. The temperature evolution was calculated during the 10-s sonication followed by a 10 s cooling period. In addition, the 2-D spatial distribution of the temperature at the end of the sonication was computed in all cases.

## Results

### Flow cytometric cell viability analysis of HT29 exposed to doxorubicin and/or hyperthermia

**Figure 3:**
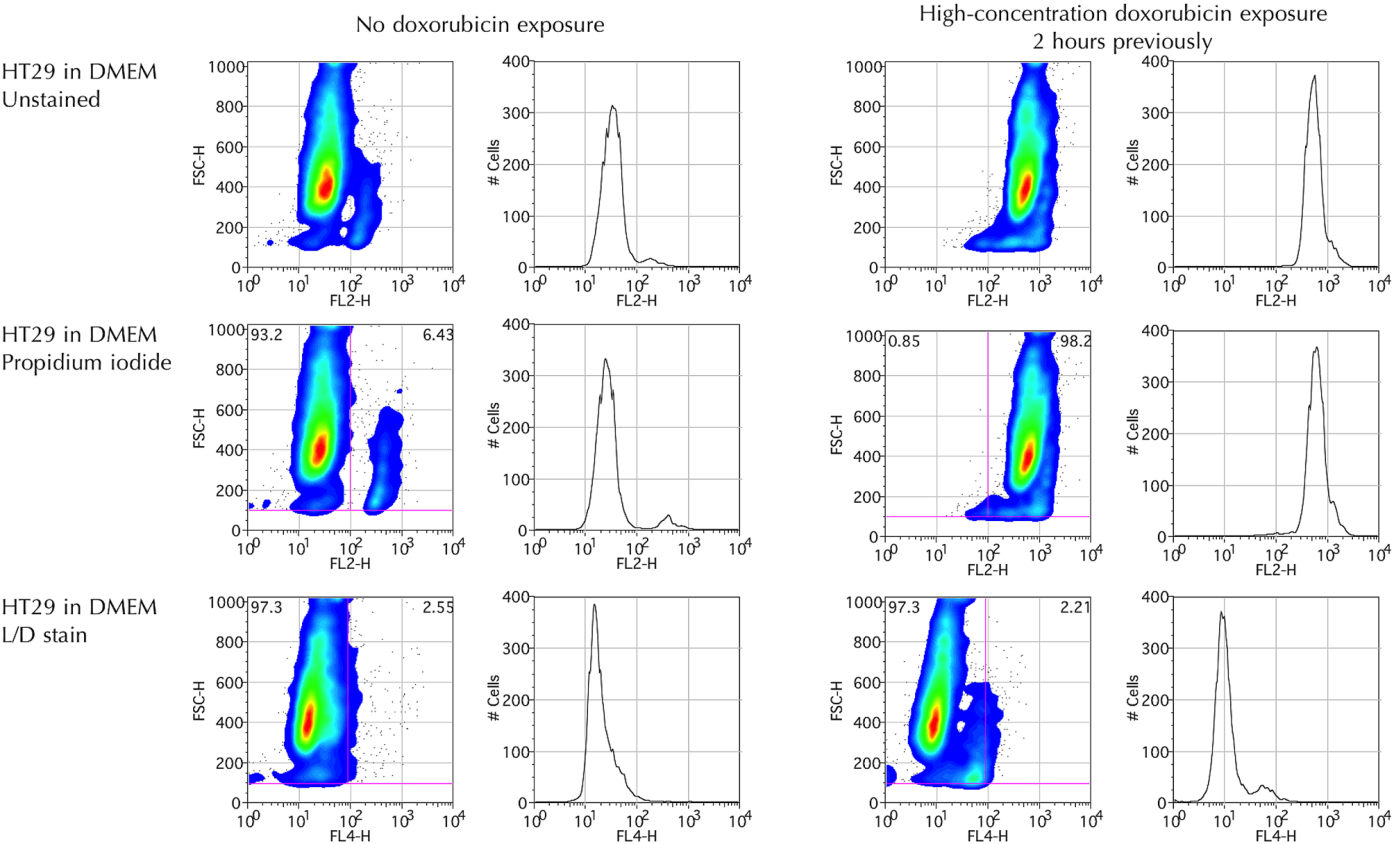
Flow cytometric studies (dot plot, histograms) of viable HT29 cell suspension exposed to high concentration doxorubicin (RIGHT) 2 h previously or not (LEFT). Samples are either unstained detected using FL2 channel (excitation/emission 488/585 ± 42 nm) (top row), stained with propidium iodide detected using the same FL2 channel (middle row), or the far-red L/D stain detected using FL-4 channel (633/660 ± 16 nm) (bottom row). On the logarithmic FL-4 scale, the vertical line represents the fluorescence gating threshold, with events to the left of the line representing viable cells, and events to the right of the line representing positive staining by the viability, i.e. what might be expected to be non-viable cells. Percentage of cell viability (top left, dot plot) vs. death (top right, dot plot) are demonstrated within the gated areas. However, note that the PI stain gives a misleading impression of near complete cell death in the only very recently doxorubicin-exposed cells due to the overlap with doxorubicin’s wide fluorescent emission profile, when in reality there has been insufficient time for true cell necrosis. Events with FSC < 100 discarded, presumed to represent debris and/or impurities, with around 90% events included for analysis. Plots generated using FlowJo Data Analysis Software.

Preliminary work demonstrated that doxorubicin did not interfere with the L/D stain emission spectra, which could clearly be distinguished by the far-red detector (660 ± 16 nm) of the flow cytometer and demonstrated absence of significant cell death at two hours with 97.5% of cells being viable, as demonstrated by only weak L/D staining (Fig. 3). When the L/D stain was substituted for propidium iodide (PI), a traditional viability stain, using the standard orange detector (585 ± 42 nm) there was a misleading impression of near-complete cell death in the doxorubicin exposed cells even at this early time point of 2 h post exposure. This is understood due to be caused by doxorubicin’s wide fluorescent emission profile interfering with the PI stain emission. The threshold levels for the L/D and PI gates were determined based on the expected findings from published literature, preliminary studies and the experimental findings.

HT29 cells were exposed to insults of either high dose doxorubicin (100 µg/mg), 30 min of 47 °C hyperthermia, both or neither (control). At 47 °C, the CEM_43_ model predicts complete cell death will ultimately ensue following just 15 min exposure of hyperthermia. Heating at this temperature for a duration of 30 min was chosen as a balance to ensure all cells would undergo complete cell death regardless of microregional fluctuations in temperature, and to avoid longer heating times at lower temperatures, which may have been an opportunity for the sample to become infected with bacteria that subsequently compromises analysis.

**Figure 4:**
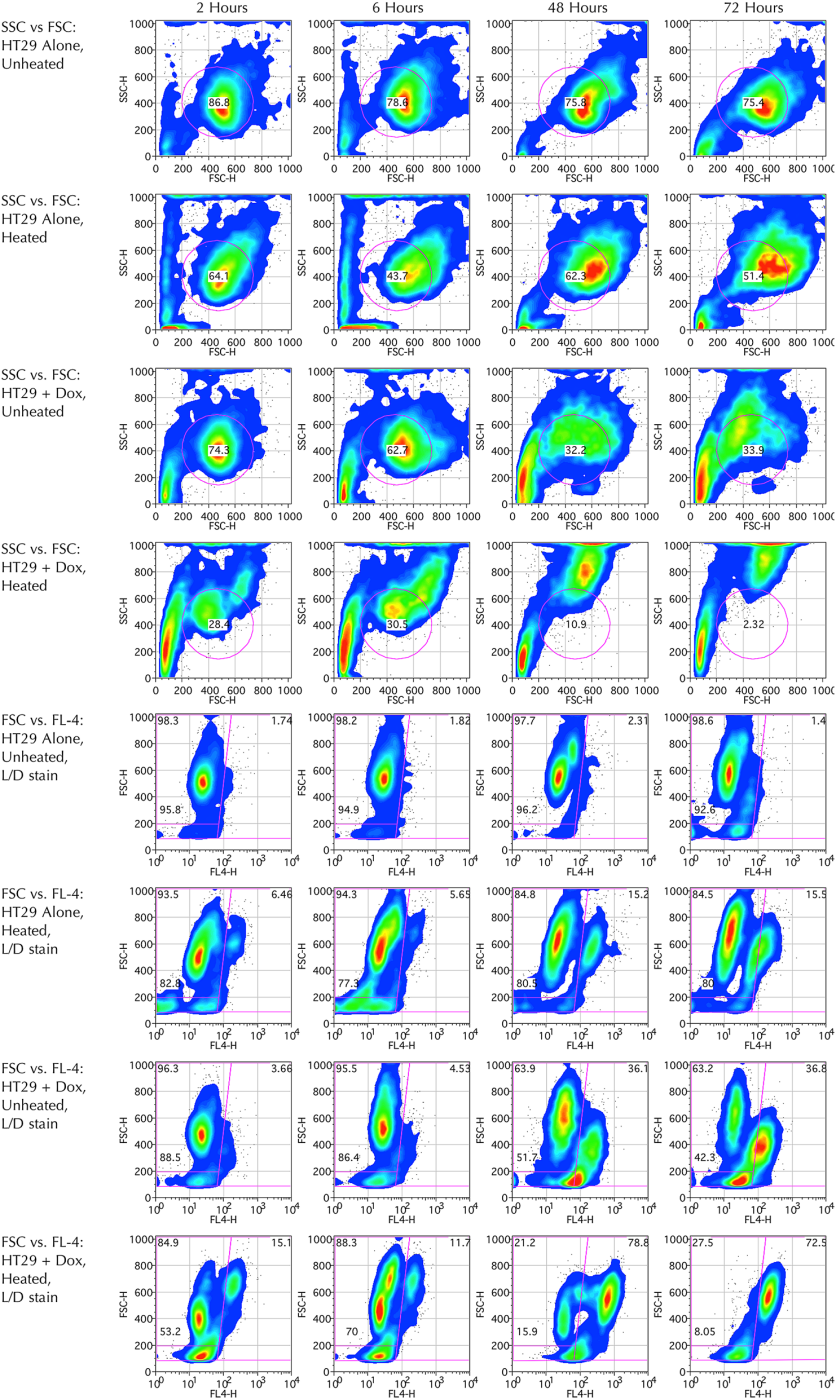
Flow cytometric studies of HT29 cell suspensions with and without 100 µg/g doxorubicin exposure and/or 30 min of 47 °C hyperthermia. Viability analysis performed using L/D stain, assayed at 2, 6, 48 and 72-h (Columns 1–4). Rows 1–4: Side Scatter (SSC) vs. Forward scatter (FSC) with population of likely viable cancer cells selected by a circle, expressed percentage of all events centrally within the circle. No events discarded. Rows 5–8: FSC vs. far-red FL-4 channel (633/660 ± 16 nm) (see methods). 10,000 events recorded and events with FSC < 100 AU discarded, presumed to represent cell debris and/or impurities. Percentage of cell viability (top left) vs. death (top right) are demonstrated within the larger (outer) gated areas. The events within the viable cell gate with FSC between 100 and 200 AU are presumed to represent apoptotic bodies, and these have been excluded using a smaller sub-gate, with percentage of all events within that gate just above the FSC = 200 AU line. Plots generated using FlowJo Data Analysis Software.

SSC, corresponding to granularity, was noted to range from approximately 200–600 AU in the majority of cells in the control sample whilst cell size (FSC) ranged from 300 to 700 AU. These cells are approximated by a circular gate encompassing 86.8% of the control cells (Fig. 4). Of note the plots demonstrate a clear perturbation in these parameters in the cells receiving insults, most notably in the doxorubicin-exposed samples with or without hyperthermia. In particular two new groups emerged; the first with a higher SSC ranging from 600 to 1000 AU (the upper limit, with some events on or close to the axis line) and slightly enlarged cell size (FSC 400–800 AU) and the second with small SSC < 400 AU and small cell size (FSC < 200 AU). These emerging groups are seen in the samples receiving insults are demonstrated as early as two hours, and are most apparent at 48 and 72-h and are likely to represent groups of early and late apoptotic cells respectively, which is described in the literature^58,59^. It is noted that due to change in FSC and SSC, only 2.3% of cells remained in the circular gate defined for the control cells on FSC/SSC axes, following both insults at 72-h. Back-gating of the circular gate on the FSC/SSC plot to the FSC/LD FL-4 (viability) axes revealed the gate contained both viable and non-viable cell populations.

**Figure 5:**
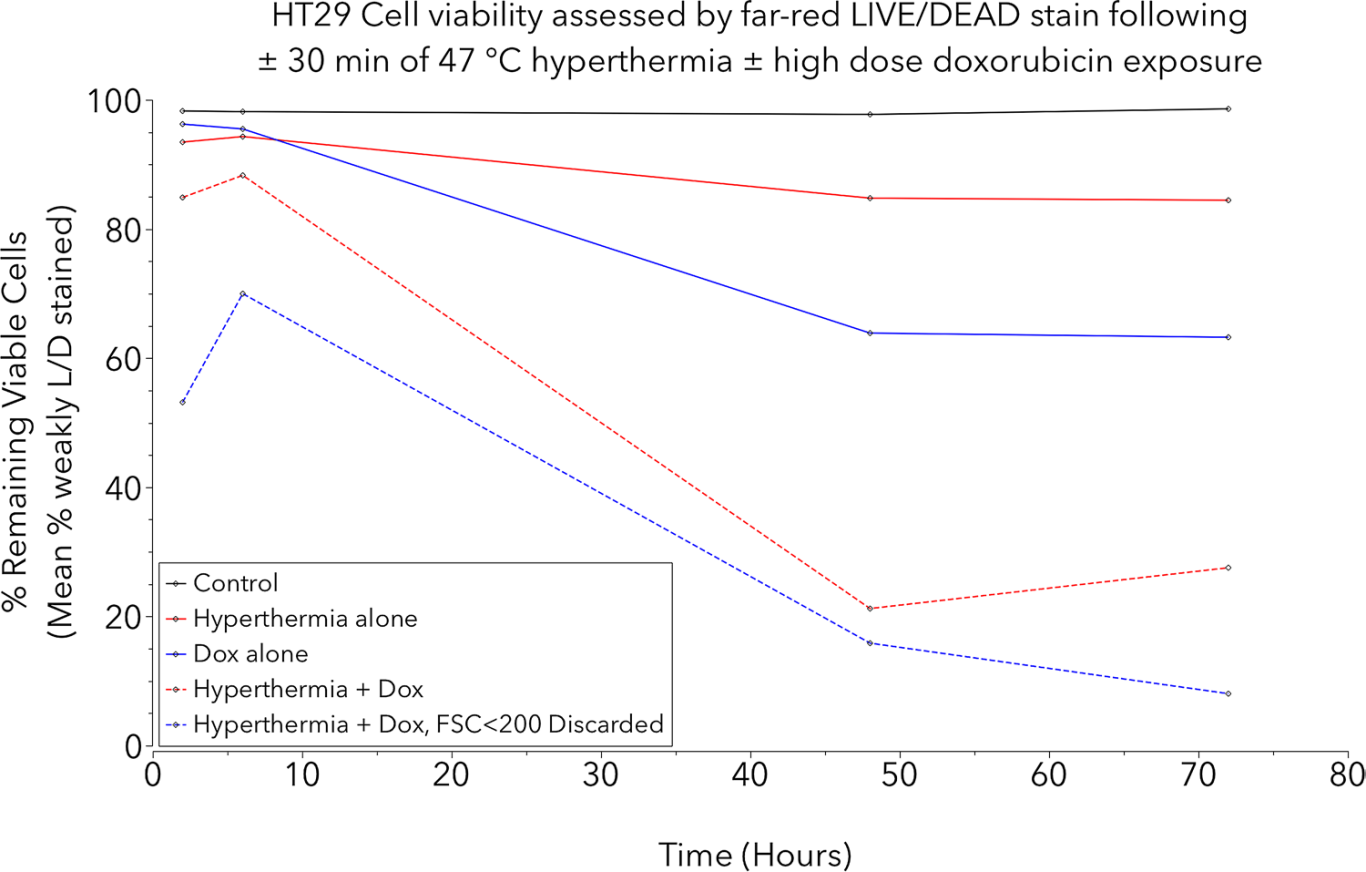
Summary plot of cell viability analysis for HT29 cells exposed to insults of either high dose doxorubicin (100 µg/mg) and/or 47 °C hyperthermia with controls, using L/D viability stain. Cells assayed at 2, 6, 24 48 and 72-h. There is more rapid and near-complete cell death in the group receiving both insults compared with the groups receiving a single insult, and minimal cell death is demonstrated in the control. Of note, if debris particles considered to be ‘apoptotic bodies’ are excluded, the results indicate near-total cell kill in the group receiving both insults at 72-h (blue dotted line).

In terms of the cell viability plots, which were stained using L/D stain, the primary gate was determined based on literature, preliminary work and finely adjusted according to the sample receiving hyperthermia alone at 48 h, where there two clear subgroups are observed: the weakly staining events to the left of the line broadly representing possible viable cells (Fig. 4). There was a clear trend for maximal cell death (strongly positive L/D stain) which was observed in the samples receiving both insults, where apparently only 27.5% of cells remained viable at 72-h compared to 98.6% in the control (Fig. 5). Crucially, with more careful interpretation of Fig. 4, the true number of viable cells is likely to be much closer to zero in the group receiving both insults at 72-h. This is because the viable cells of the control group at two hours (first row, first column) have FSC over 200, yet the group exposed to both insults at 72-h (fourth row, forth column) demonstrates near-absence of events in the viable gate with FSC over 200, with a cluster of events with FSC between 100 and 200. These events are likely to represent cells that have fragmented into apoptotic bodies, rather than true viable cells, thus falsely over-representing the true percentage of viable cells^60^. With exclusion of these apoptotic bodies, analysis reveals 92% cell kill at this timepoint, which is more in line with what might be expected from the lethal combination of these high severity insults. This result highlights the importance of meticulous gating in flow cytometry and the potential for result misinterpretation.

### Quantification of ex vivo liver cell recovery

During optimisation of the cell recovery protocol in control ex vivo bovine liver samples, it was determined that the protocol recovered an average of over 3000 cells per mg tissue for processed tissue masses ranging from 2 to 22 mg.

### Flow cytometric cell viability analysis of ex-vivo liver tissue exposed to hyperthermia alone

Throughout the treatment period, thermometry traces revealed mean temperatures of 44.0 °C and 37.4 °C in the hyperthermic and normothermic water baths respectively.

**Figure 6:**
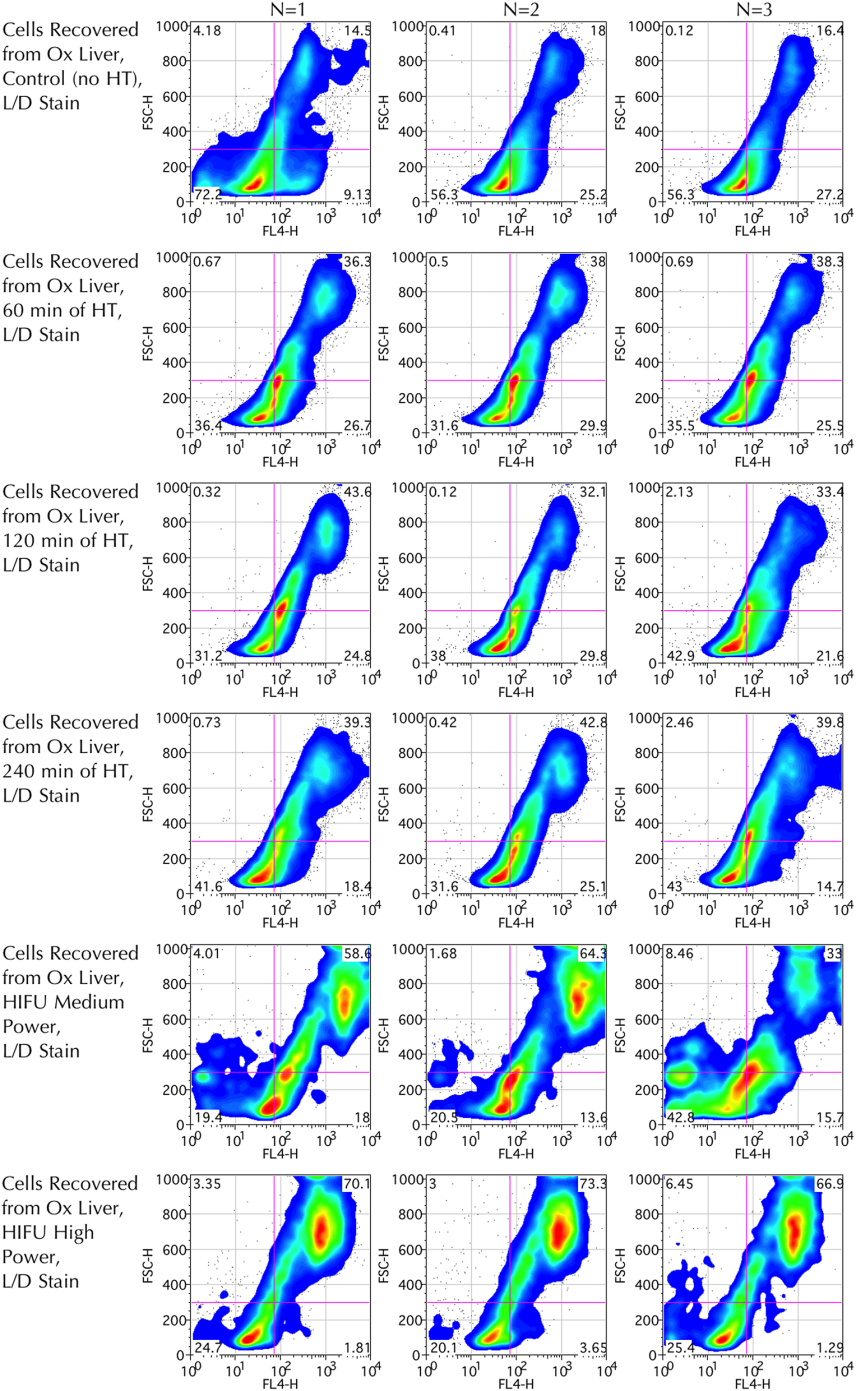
Cell viability flow cytometric studies of cells recovered from control liver and liver exposed to varying durations of hyperthermia and powers of HIFU. Each condition was performed in triplicate, i.e. three bags with liver sample exposed to hyperthermia and three liver lesions were performed and sampled individually at each HIFU power (N = 3, Columns). Representative plots for 0, 60, 120 and 240 min of hyperthermia (rows 1–4) and medium (7.27 MPa) and high (8.19 MPa) HIFU powers (rows 5–6) shown using FSC vs. far-red FL-4 channel (633/660 ± 16 nm). Events with FSC < 70 and/or FL-4 < 300 discarded, presumed to represent cell debris and/or impurities. Viability assessed using L/D stain. Trend for increased L/D staining intensity with samples receiving increasing insults, most markedly in the high power HIFU group, compared to the control group. Plots generated using FlowJo Data Analysis Software.

Flow cytometric plots revealed that recovered ex vivo liver cells in the control group, mainly comprising of healthy hepatocytes, are smaller (FSC of around 70–300, Fig. 6), than the control HT29 cells (FSC 300–700 AU, Fig. 4). For all flow cytometric analysis performed on ex vivo bovine liver, events with FSC < 70 AU and/or L/D staining (FL-4) < 300 AU were discarded to remove cellular debris, which was of particular importance to the later HIFU-treated group.

**Figure 7:**
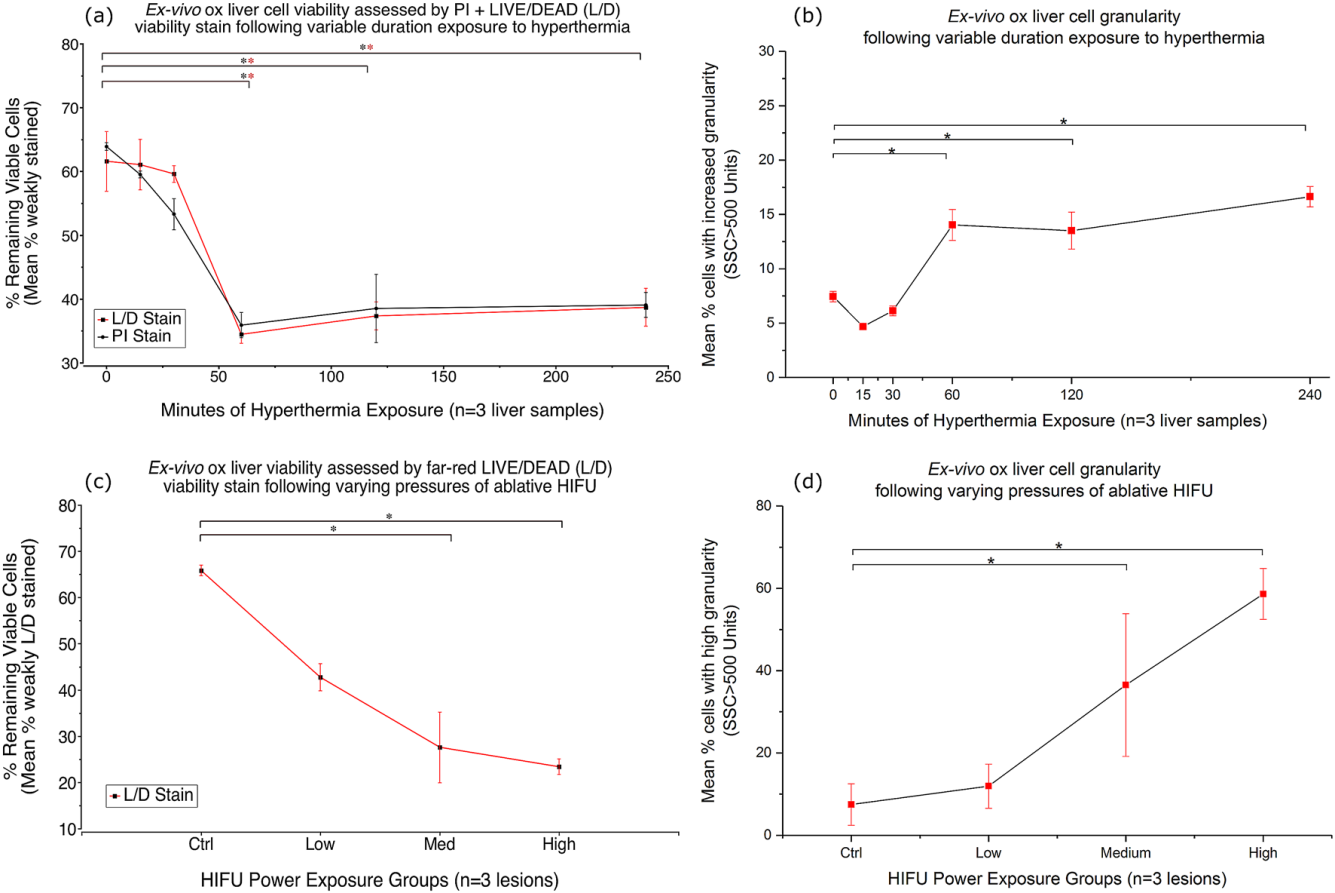
Cell viability analysis for ex vivo bovine liver experiments. (**a**) Mean cell viability plot of cells recovered from control liver and liver exposed to varying durations of hyperthermia (45 °C) assayed at 16 h (N = 3). One-way ANOVA with Fisher’s LSD revealed significant increase in cell death (p < 0.01) for the groups treated with 60, 120 and 240 -min of hyperthermia when compared to the control, 15- and 30-min groups in any combination for both PI and L/D stains (**). (**b**) Cell granularity analysis for the same (hyperthermia) group (N = 3). One-way ANOVA with Fishers LSD revealed a significant increase (p < 0.01) in granularity (SSC) for the 60-to-240-min hyperthermia exposure groups when compared to the control. (**c**) Mean cell viability plot of cells recovered from control liver and HIFU-ablated lesions fixed at 4 h and assayed the following day (N = 3). One-way ANOVA with Fisher’s LSD revealed significant increase in cell death (p < 0.01) in both the medium and high-power groups compared to both the control group in any combination for the L/D stain (*). (**d**) Cell granularity analysis for the same (HIFU) group (N = 3). One-way ANOVA with Fishers LSD revealed a significant increase (p < 0.01) in granularity (SSC) for the medium and high HIFU exposure groups when compared to the control.

At 16 h after exposure to hyperthermia for an hour or more, a statistically significant (p < 0.01) increase (1.67-fold) in cell death (strong L/D staining) was evident compared to the control group as well as the two groups treated for 15 or 30-min (Figs. 6, 7). Statistical analysis revealed that, compared to the control group, there was a statistically significant increase in granularity (p < 0.01) as measured by SSC^73^, (with SSC > 500 used as the threshold) in the groups exposed to hyperthermia for 60-min or more (Fig. 7).

### Flow cytometric cell viability analysis of ex-vivo liver tissue exposed to HIFU ablation

It was noted that cell recovery was poorer in the HIFU-ablated samples and on average, across the six lesions, 9097 events where captured (of an attempted 10,000 events), with around 7593 events remaining following exclusion of debris (Supplementary Table S1). This was in contrast to the cell recovery for control and hyperthermia exposed liver samples, where the attempted 20,000 events were recovered in every case and cell counts were likely in high excess of this. This is not unexpected and is likely due to instantaneous cell destruction caused by HIFU, as oppose to apoptotic mechanisms in hyperthermia which may preserve the basic cell structure for longer before death ensues.

**Figure 8:**
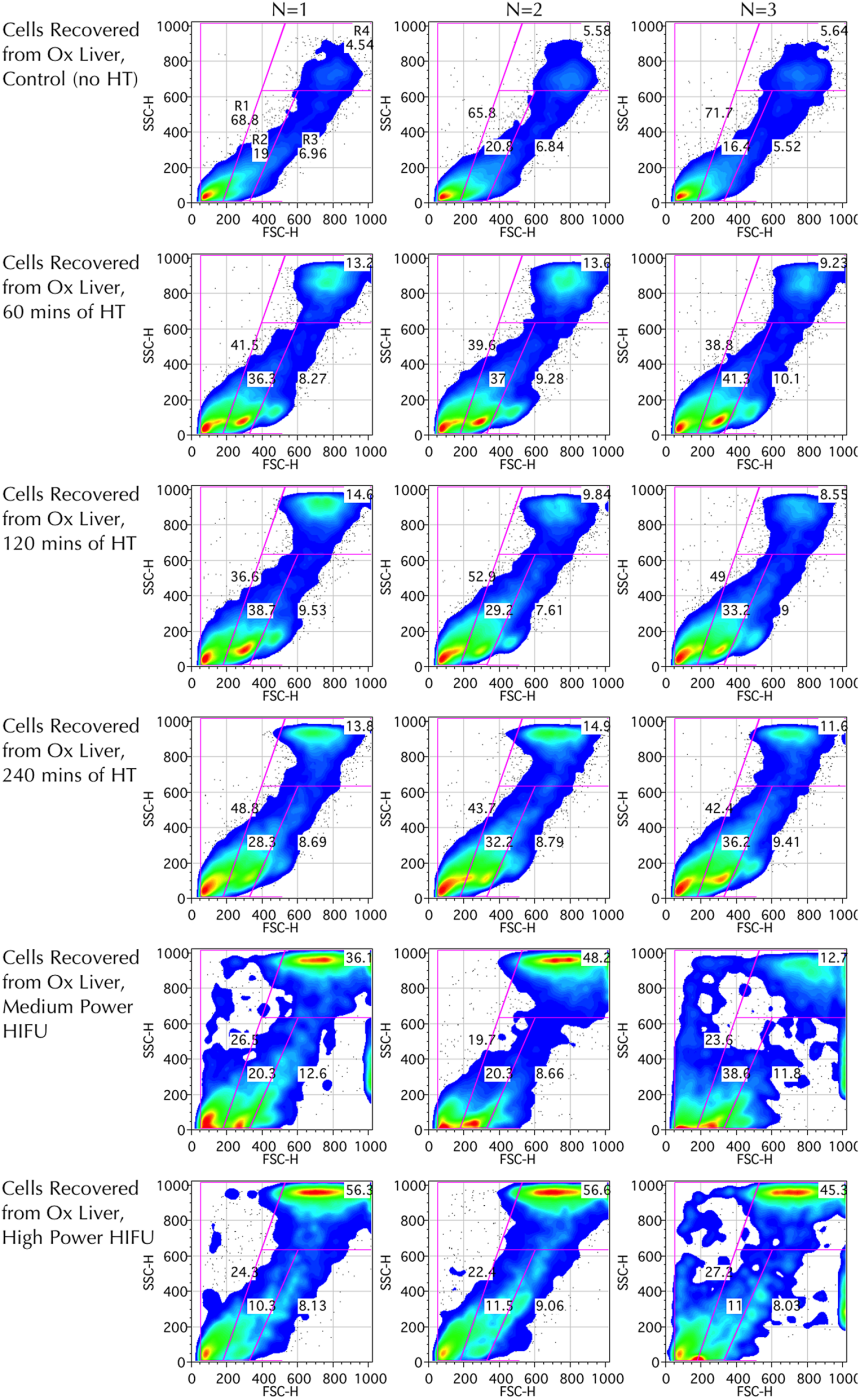
SSC and FSC flow cytometric studies of cells recovered from control liver and liver exposed to varying durations of hyperthermia and powers of HIFU (N = 3, Columns). Representative plots for 0, 60, 120 and 240-min of hyperthermia (rows 1–4) and medium (7.27 MPa) and high (8.19 MPa) HIFU powers (rows 5–6) shown. Sub-populations have been gated, with gates labelled in top-left plot only. Plots demonstrate a trend for at first increased FSC (moving from R1 to R2), followed by dramatically increased SSC and FSC (cells moving from R1/R2 populations to R4) with increasing severity of hyperthermic or ablative insults, consequent with larger cell size and granularity. Events with FSC < 70 discarded. Plots generated using FlowJo Data Analysis Software.

Following hyperthermic or HIFU insult, flow cytometric analysis revealed that some bovine liver cells increased moderately in size with only modest increase in SSC, moving from the R1 to the R2 and R3 gated populations (Fig. 8). These R2-R3 populations are thought to represent increased cellular homogeneity and size due to early apoptotic mechanisms. Although apoptosis is not thought of as being classically associated with HIFU, it may be seen in surrounding areas receiving insult which are not immediately necrosed^74^, and it is conceivable that the tissue sampling included a small margin liver exposed to sub-ablative HIFU levels. Late apoptotic cells, which are small, condensed cells with low FSC and low SSC, are not seen and this is likely due to being excluded along with cell debris (FSC < 70 AU) given the small initial cell size of the hepatocytes. Other cells demonstrate a more dramatic increase in both SSC and FSC, moving into the R4 population, which is particularly prominent in the HIFU-treated groups. This group is thought to represent swollen necrotic cells, with gross internal inhomogeneity, which have ensued cell death through non-apoptotic mechanisms. Of note, back-gating revealed that almost all events in the R1 population demonstrate weak L/D staining (thus are viable), whilst R2 population is mostly viable and the R3 and R4 populations are almost all non-viable. Conversely, when back-gating from the viability plot, the top right gate of high SSC and high FSC correlated to events in both R2, R3 and R4, whilst the remaining three gates correlated to R1 and R2 populations.

Statistical analysis revealed that, compared to the control group, there was a statistically significant increase in both granularity (p < 0.01) as measured by SSC^73^, (with SSC > 500 used as the threshold) and cell death (p < 0.01) as measured by strongly positive L/D staining in the groups exposed to medium and high pressure HIFU (Fig. 7). Overall, means of 7.4% vs. 58.6% for high granularity and 23.4% vs. 65.8% for L/D viability were demonstrated in the high HIFU pressure and control groups respectively. Viability using the L/D stain correlated closely with the PI stain, in the absence of doxorubicin (Fig. 7).

It is likely that the percentage of remaining viable cells measured post-HIFU are an overestimate due to over-selection of intact cells by flow cytometry when in fact most of the cells have been destroyed and become cell debris, much of which is discarded during centrifuge steps. This is supported by the poor cell yields in the HIFU cases; under 10,000 events were captured in 3 of 6 samples (Supplementary Table S1). In reality it is suspected that there is near-complete cell death in the HIFU-treated groups (see “Limitations of study” section).

### Immunohistochemical analysis of ex-vivo liver tissue exposed to HIFU ablation

**Figure 9:**
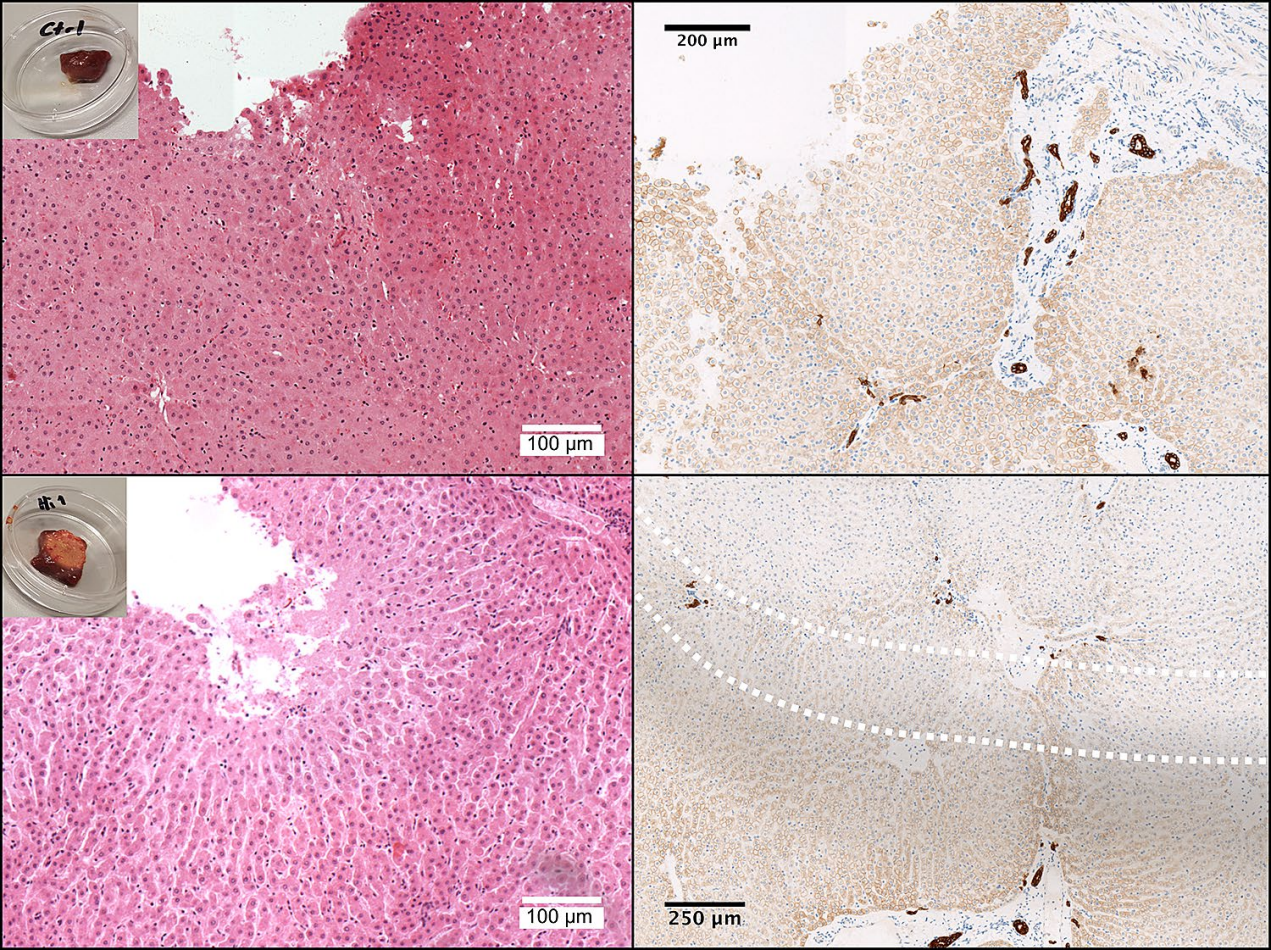
Immunohistochemistry of HIFU-ablated *ex-vivo* bovine liver exposed to 8.2 MPa peak-to-peak. H&E (left column) and pan-CK microscopy at ×20 of control sample (top row) and HIFU-exposed sample (bottom row). Insets of macroscopic samples shown in the top left corner of H&E images. Microscopy was taken at lesion edge which was clearly defined in the case of pan-CK but less easily distinguished with H&E. H&E of the HIFU-ablated liver demonstrates distinct cellular margins, subtle loss of some nuclear stain and occasional haemorrhagic foci. The dotted lines in the HIFU-ablated pan-CK sample (bottom right) demonstrate an approximate 300 µm transition zone of some viable hepatocytes between viable cells (below, positive staining) and non-vital hepatocytes (above, negative staining). The bile ducts are noted to stain strongly, regardless of HIFU exposure (dark brown).

H&E microscopy of the control and HIFU-exposed samples revealed the ablated liver architecture appears relatively preserved, although there was a distinct cellular margin, subtle loss of staining of some of the nuclei, in comparison to the control H&E and small haemorrhagic foci (Fig. 9). The changes to the cellular margin are thought to be due to desiccation and heat-fixation of cells. Findings are consistent with published data on HIFU ablation of tumour cells in human solid malignancies, although not as pronounced^28^, and not too dissimilar to findings of a published study using in porcine liver at 2 h post ablation with irreversible electroporation^75^. Due to harvest and fixation so shortly (within 1 h) post-HIFU ablation, it is thought that the morphological changes demonstrated on H&E in Fig. 9 are very early, hence there is only subtle loss of nuclear stain in comparison to the gross loss with the clinical study in which tumour tissue was obtained 1–7 days post-HIFU^28^. The early timing would also explain the lack of infiltrating inflammatory cells and fibroblasts seen clinically at 7 days post-HIFU and complete cellular breakdown seen at 10–14 days. Pan-CK immunohistochemistry revealed that the control hepatocytes are weakly stained, consistent with the liver being an epithelial derivative (of the foregut) (Fig. 9). Bile ducts demonstrate a very strong stain, as would be expected for true epithelial structures^76^ and has previously been reported^77^. In the HIFU-treated samples, loss of pan-CK staining of the hepatocytes in the ablated zone was clearly demonstrated, correlating macroscopically, and there was a 200–300 µm transition zone at the well-defined ablation margin. Staining of the bile-ducts appears subtly weaker in HIFU-exposed samples but overall appear relatively unaffected. Similar loss of pan-CK stain was demonstrated in samples exposed to a lower HIFU power of 6.34 MPa peak-to-peak, albeit over smaller areas for the same HIFU duration (data not shown). Crucially the pan-CK stain not only more clearly differentiates the HIFU-ablated zone, due to its mechanism, it does so near-instantaneously in tissue which has ablated, regardless of modality^30^.

### Simulation results

**Figure 10:**
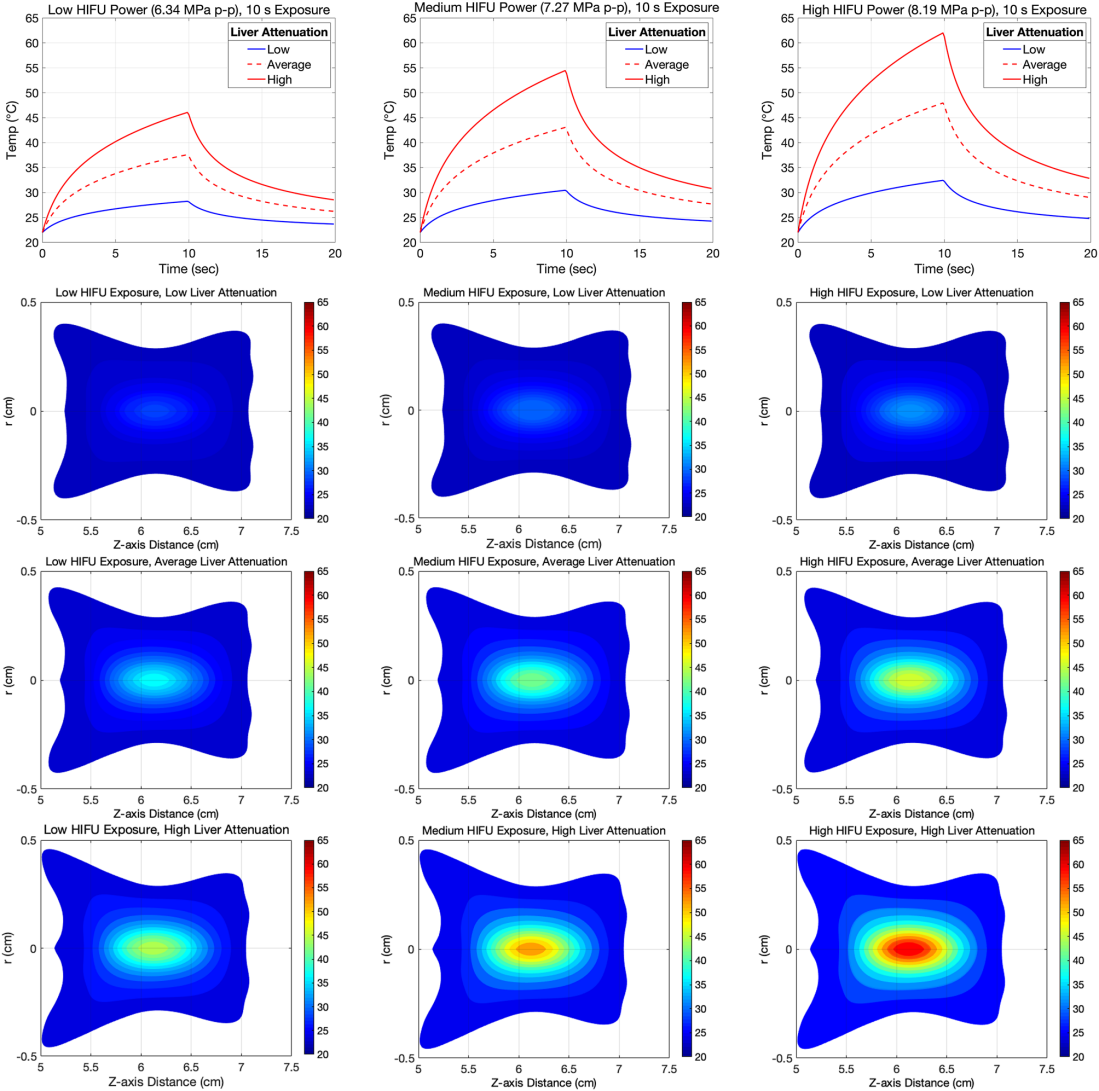
Simulations of expected temperature rises obtained in the *ex-vivo* liver tissue performed using non-perfused and non-linear KZK model. TOP: Temperature plots were simulated for a single continuous wave 10 s sonication at the transducer’s focal depth of 51.7 mm penetrating 20 mm into tissue. The solid red curve represents high liver attenuation, the dotted red curve average liver attenuation and the blue, low liver attenuation. BOTTOM (Rows 2–4): Temperature maps were simulated using the same parameters at low (row 2), average (row 3) and high (row 4) liver attenuation. Left Column: 6.34 MPa, Middle Column: 7.27 MPa, Right Column: 8.19 MPa peak-to-peak acoustic pressures.

Simulations of the FUS field demonstrated non-overlapping regions of hyperthermia using 1 cm separation between lesions in the XY plane, as the maximum radial extent for all lesions was demonstrated to be under 0.5 cm (Fig. 10). However, by design, the 2 × 2 grid of sonications that formed each of the nine lesions had some degree of overlap due to having only 1 mm dot separation. Simulations represent a single sonication in the 2 × 2 grid, not all four sonications.

The measured temperature plots demonstrate that at parameters for average liver attenuation, the peak temperature attained at the highest experimental HIFU pressure used (8.19 MPa peak-to-peak) was 47 °C from a baseline of 22 °C, whereas at the parameters for highest liver attenuation, a peak temperature of 62 °C was attained. Thermal dose simulation, based on a single sonication per lesion, revealed that the only combination to exceed the CEM_43_ threshold was at the highest HIFU exposure and highest liver attenuation. Based on practical experience from preliminary experiments using this experimental set up, ablation zones (lesions) were clearly visible macroscopically even at the medium HIFU exposure pressure (7.27 MPa peak-to-peak free-field, 6.6–7.54 MPa derated), presumably due to non-thermal and mechanical effects of HIFU, whilst remaining under the thermal dose threshold^23^. Previous preclinical studies have shown that at 1 MHz frequency, the inertial cavitation threshold is around 4–6 MPa peak pressure (~ 8–12 MPa peak-to-peak)^78–80^. These low, medium and high HIFU pressure exposure parameters were thus chosen in an attempt to differentiate sub-ablative levels of cell death at the lower pressures and complete ablation at the highest pressure for the 10 s continuous wave exposures (with some overlap within the lesion grid). It is anticipated that higher pressures would be required to induce ablative lesions in vivo, for example due to effects of tissue perfusion. Indeed, in the TARDOX study, mild volumetric liver hyperthermia was induced at 1 MHz using upper-bound estimate peak-to-peak in situ (derated) pressures in the range of 10–16 MPa, at or just above the inertial cavitation threshold. Despite this, constant transducer movement required for volume painting meant much reduced dwell time in a particular spot, and CEM_43_ was calculated or predicted below the thermal dose threshold. This was supported clinically by the absence of histological or radiological evidence of thermal ablation post-FUS exposure^12^ (Supplementary Material Table S1).

## Limitations of study

- The method has been validated in healthy ex vivo hepatocytes and a tumour cell line exposed to heat and drug insults but has not been validated in clinical tumour samples.
- Whilst the study looks at the use of FSC/SCC plots for identification of apoptotic cell populations, the method presented does not include specific stains for apoptotic markers. For example using the annexin-V stain which will stain apoptotic cells before the plasma membrane becomes permeable to PI at point of cell necrosis^58^. The methodology presented is easily amenable to include such apoptotic stains, depending on the requirements of the study.

## Limitations of flow cytometric methodology

- Flow cytometry of cells obtained from tissue disaggregation is consequent with loss of tissue morphology, which is readily available from microscopic studies. For example, the distribution of cellular doxorubicin in relation to tumour vessels can only be obtained with fluorescence microscopy^81^. In some cases, depending on available tissue masses, a combination of both flow cytometric and histopathological analysis may be of value.
- Single biopsies may not be fully representative of a heterogenous tumour environment; sampling in the necrotic core of a tumour is likely to give markedly different results to a peripheral sample.
- With any flow cytometric study, an understanding of the cell populations and careful attention to discard and gating thresholds are crucial to correctly interpret data, and will naturally vary depending on cell type, their exposures and stains used. In this study, rather than using automated gating methodology, gates were determined in knowledge of the expected thresholds for the stains, with experience of the cell population being analysed followed by fine tuning by eye based on the resulting data. This is illustrated in Fig. 4 where the percentage viability of HT29 cells exposed to both hyperthermic and doxorubicin insults is reduced from 27.5% to a more realistic 8% once apoptotic bodies are gated out. However, caution is recommended as setting the FSC discard threshold too high, as important data may be excluded, for example the control ex vivo liver cells are smaller (FSC 70–300) than the control HT29 cells (FSC 300–700), making it difficult or impossible to distinguish apoptotic bodies without further staining.
- There is potential for flow-cytometry to over-select intact cells and to ignore cell fragments which may in fact be representative of dead cells when using other techniques such as microscopy. This bias is introduced from both the inability to capture tiny cell fragments discarded after centrifuging during the cell recovery process and the gating issues described. This may be especially important in the ablative HIFU groups; it is likely that the resulting mean of 23.4% viable ex vivo liver cells following high power HIFU is an overestimate due to over selection of cellular events, whilst the apoptotic bodies and other cell fragments with FSC < 100 AU which do make it through centrifuge are indiscriminately discarded. In reality the proportion of cell death is likely near-complete in this group.

Regardless of these pitfalls, flow cytometry has advantages of being a highly quantitative, practical, versatile and high throughput technique in comparison to more qualitative assessments by microscopy.

## Discussion

We have demonstrated that, with optimised cell recovery techniques, flow cytometry offers a rapid, sensitive and highly quantitative method to assess cell viability in typical masses obtained from liver biopsy cores of 14-18 Gauge (G) (5–20 mg)^52^. Results have been correlated with alternative immunohistochemical microscopic techniques, demonstrating clear ablative change in heavily ablated non-pathological ex vivo liver tissue, but this method may be more subjective in a heterogenous tumour environment at sub-ablative or near-ablative thresholds.

With continued advancements in thermally sensitive drug delivery systems, such as LTSLs^5,6,11,82^, real-time monitoring capabilities of clinical HIFU devices^83^ and the potential for predictive hyperthermia models using cross-sectional imaging data^13^, hyperthermia-mediated targeted drug delivery using FUS is gaining momentum^12^. It is critical that safety of the hyperthermia regime is evaluated in the early clinical studies; FUS parameters must be optimised to achieve localized hyperthermia compatible with targeted drug-delivery in the absence of ablation. Whilst real-time in silico modeling of the CEM_43_ thermo-tolerance model is used by devices clinically, it is not validated for human tissues or the large, instantaneous temperature rises seen in ablative HIFU and therefore has inherent limitations. In early phase or proof of concept clinical studies, whilst biopsies may be primarily required for quantifying drug delivery endpoints, they may also provide a wealth of quantitative information relating to cell viability and immunological markers. The method presented may be used to demonstrate FUS exposures to tissues for mild hyperthermia, for example as used in drug delivery of thermosensitive liposomes, are below the ablative threshold.

## Conclusion

We have presented a robust, sensitive and highly quantitative cell recovery and flow-cytometric cell viability method, which has been validated in ex vivo liver tissue exposed to ablative and sub-ablative levels of FUS and isolated colorectal cancer cell line studies involving hyperthermia and doxorubicin insults. This method is applicable for biopsy samples received from study participants undergoing targeted delivery of doxorubicin using thermo-sensitive drug delivery systems (such as LTLD) in combination with FUS or other heating modalities. The methodology could be extended to examine other cell attributes, such as apoptotic status or immunostaining for relevant disease or treatment response markers, and is adaptable for analysis of tissues exposed to other chemotherapeutics.

## Supplementary Information

Supplementary Information.

## Data Availability

The datasets during and/or analysed during the current study available from the corresponding author on reasonable request.

## Abbreviations

ANOVA: Analysis of variance
BHT: Pennes’ bioheat transfer equation
CEM_43_: Cumulative equivalent in minutes at 43 °C
DMEM: Dulbecco’s modified eagle medium
DMSO: Dimethyl sulfoxide
DPBS: Dulbecco’s phosphate buffered saline
FACS: Fluorescence-activated cell sorting
FSC: Forward scatter
FUS: Focused ultrasound
HIFU: High intensity focused ultrasound
H&E: Haematoxylin and eosin
HPLC: High performance liquid chromatography
L/D stain: Far Red LIVE/DEAD fixable far red dead cell stain kit
LTLD: Lyso-thermosensitive liposomal doxoroubicin
LTSL: Liposomal thermosensitive liposomes
LSD: Least significant difference
MRgFUS: MRI-guided focused ultrasound
Pan-CK: Pan-cytokeratin
PI: Propidium Iodide
RMPI: Roswell Park Memorial Institute medium
TID: Thermal isoeffect dose
SSC: Side scatter
USgFUS: Ultrasound-guided focused ultrasound
KZK: Khokhlov–Zabolotskaya–Kuznetsov equation
