# Supplementary material for: Quantifying cell death induced by doxorubicin, hyperthermia or HIFU ablation with flow cytometry
Source: Sci Rep. 2021 Feb 23;11:4404. doi: 10.1038/s41598-021-83845-2 (PMC7902827; doi:10.1038/s41598-021-83845-2)
Supplement: Supplementary file 1 — Supplementary Information. [file 41598_2021_83845_MOESM1_ESM.docx]

**QUANTIFYING CELL DEATH INDUCED BY DOXORUBICIN, HYPERTHERMIA OR HIFU ABLATION WITH FLOW CYTOMETRY**

**Paul Christopher Lyon^1,2^, Visa Suomi^2^, Philip Jakeman^3^, Leticia Campo^3^, Constantin Coussios^2^, Robert Carlisle^2^**

^1^Nuffield Department of Surgical Sciences, University of Oxford, John Radcliffe Hospital, Oxford OX3 9DU

^2^Institute of Biomedical Engineering, Old Road Campus Research Building, University of Oxford, Oxford OX3 7DQ

^3^Department of Oncology, University of Oxford, Old Road Campus Research Building, University of Oxford, Oxford, OX3 7DQ

**Corresponding Author:**

Paul Lyon: lyon.hifu@gmail.com

**Short Running Title:**

Quantification of Cell Death by Doxorubicin, Hyperthermia or HIFU by Flow Cytometry

**SUPPLEMENTARY MATERIAL**

| **Experiment Description** | **Details** | **Events captured** | **Events remaining after discard** |
| --- | --- | --- | --- |
| Figure 3, HT29 ± Dox, N=1 | No dox, unstained | 10000/10000 | 9368/10000 |
|  | No dox, PI | 10000/10000 | 9700/10000 |
|  | No dox, L/D | 10000/10000 | 9742/10000 |
|  | High-conc. dox, unstained | 10000/10000 | 9381/10000 |
|  | High-conc. dox, PI | 10000/10000 | 9453/10000 |
|  | High-conc. dox, L/D | 10000/10000 | 9372/10000 |
| Figure 4, HT29 + Dox + HT  FL4=L/D | No dox, unheated, 2 hr | 10000/10000 | 9764/10000 |
|  | No dox, unheated, 6 hr | 10000/10000 | 9609/10000 |
|  | No dox, unheated, 48 hr | 10000/10000 | 9853/10000 |
|  | No dox, unheated, 72 hr | 10000/10000 | 9706/10000 |
|  | No dox, heated, 2 hr | 10000/10000 | 8932/10000 |
|  | No dox, heated, 6 hr | 10000/10000 | 8862/10000 |
|  | No dox, heated, 48 hr | 10000/10000 | 9653/10000 |
|  | No dox, heated, 72 hr | 10000/10000 | 9603/10000 |
|  | Dox, unheated, 2 hr | 10000/10000 | 8960/10000 |
|  | Dox, unheated, 6 hr | 10000/10000 | 8784/10000 |
|  | Dox, unheated, 48 hr | 10000/10000 | 7337/10000 |
|  | Dox, unheated, 72 hr | 10000/10000 | 7745/10000 |
|  | Dox, heated, 2 hr | 10000/10000 | 6906/10000 |
|  | Dox, heated, 4 hr | 10000/10000 | 8003/10000 |
|  | Dox, heated, 4 hr | 10000/10000 | 8491/10000 |
|  | Dox, heated, 4 hr | 10000/10000 | 7797/10000 |
| Figure 8, *ex vivo* bovine liver + 60,120,240 mins of hyperthermia or Medium (7.27 MPa) or High Power (8.19 MPa) HIFU, N=3  FL4=L/D | Control, n=1 | 20000/20000 | 19605/20000 |
|  | Control, n=2 | 20000/20000 | 19940/20000 |
|  | Control, n=3 | 20000/20000 | 19949/20000 |
|  | 60min HT, n=1 | 20000/20000 | 19947/20000 |
|  | 60min HT, n=2 | 20000/20000 | 19946/20000 |
|  | 60min HT, n=3 | 20000/20000 | 19910/20000 |
|  | 120min HT, n=1 | 20000/20000 | 19954/20000 |
|  | 120min HT, n=2 | 20000/20000 | 19959/20000 |
|  | 120min HT, n=3 | 20000/20000 | 19943/20000 |
|  | 240min HT, n=1 | 20000/20000 | 19940/20000 |
|  | 240min HT, n=2 | 20000/20000 | 19980/20000 |
|  | 240min HT, n=3 | 20000/20000 | 19967/20000 |
|  | Med HIFU, n=1 | 5895/10000 | 5507/5895 |
|  | Med HIFU, n=2 | 10000/10000 | 9768/10000 |
|  | Med HIFU, n=3 | 9255/10000 | 8109/9255 |
|  | High HIFU, n=1 | 10000/10000 | 9827/10000 |
|  | High HIFU, n=2 | 10000/10000 | 9873/10000 |
|  | High HIFU, n=3 | 4470/10000 | 3579/4470 |

Table S1: Number of events captured for the flow cytometry plots within the manuscript. For HT29 cell culture, events with FSC<200 removed, presumed to represent debris. For *ex vivo* bovine liver cells post tissue recovery, which are smaller than HT29 cells, events with FSC<70 and/or FL4<300 removed, presumed to represent debris.
